# Pathogenesis of Acquired Aplastic Anemia and the Role of the Bone Marrow Microenvironment

**DOI:** 10.3389/fonc.2018.00587

**Published:** 2018-12-05

**Authors:** Michael Medinger, Beatrice Drexler, Claudia Lengerke, Jakob Passweg

**Affiliations:** ^1^Division of Internal Medicine, Department of Medicine, University Hospital Basel, Basel, Switzerland; ^2^Division of Hematology, Department of Medicine, University Hospital Basel, Basel, Switzerland

**Keywords:** aplastic anemia, microenvironment, microvessel density, mesenchymal stem cells, stem cell niche

## Abstract

Aplastic anemia (AA) is characterized by bone marrow (BM) hypocellularity, resulting in peripheral cytopenias. An antigen-driven and likely auto-immune dysregulated T-cell homeostasis results in hematopoietic stem cell injury, which ultimately leads to the pathogenesis of the acquired form of this disease. Auto-immune and inflammatory processes further influence the disease course as well as response rate to therapy, mainly consisting of intensive immunosuppressive therapy and allogeneic hematopoietic cell transplantation. Bone marrow hematopoietic stem and progenitor cells are strongly regulated by the crosstalk with the surrounding microenvironment and its components like mesenchymal stromal cells, also consistently altered in AA. Whether latter is a contributing cause or rather consequence of the disease remains an open question. Overall, niche disruption may contribute to disease progression, sustain pancytopenia and promote clonal evolution. Here we review the existing knowledge on BM microenvironmental changes in acquired AA and discuss their relevance for the pathogenesis and therapy.

## Introduction

Acquired aplastic anemia (AA) is characterized by a hypoplastic, fatty bone marrow (BM) with profound reductions in hematopoietic stem/progenitor cells (HSCs/HPCs) that lead to defective mature blood cell production and peripheral pancytopenia (1–3). Diagnosis of AA requires per definition at least two of the following criteria: Hemoglobin <100 g/L, platelets <50 G/L and neutrophils <1.5 G/L, together with a hypocellular BM and in the absence of abnormal infiltrates or fibrosis (4). The description of AA as an “empty” BM in which hematopoietic cells have been replaced by fat cells was first made by Paul Ehrlich (5). Nowadays, AA is defined by decreased numbers of BM HSCs and HPCs resulting in a hypo- or aplastic BM with precocious fat replacement (1, 4).

AA can be inherited or acquired (1, 6). All patients—regardless of etiology—typically present with anemia symptoms, bleeding, and infections (7, 8). Onset at young age, additional pathologies and/or positive family history may indicate congenital or inherited syndromes driven by genetic alterations. Several inherited bone marrow failure (BMF) syndromes have been described, among which the most common are Fanconi anemia, Dyskeratosis Congenita, Diamond Blackfan anemia, and Shwachman-Diamond syndrome. The pathogenesis of these genetic conditions has been extensively reviewed elsewhere (6) and is different from that of acquired AA, which is of considered of immunological nature and is the focus of the current review.

**Figure 1:**
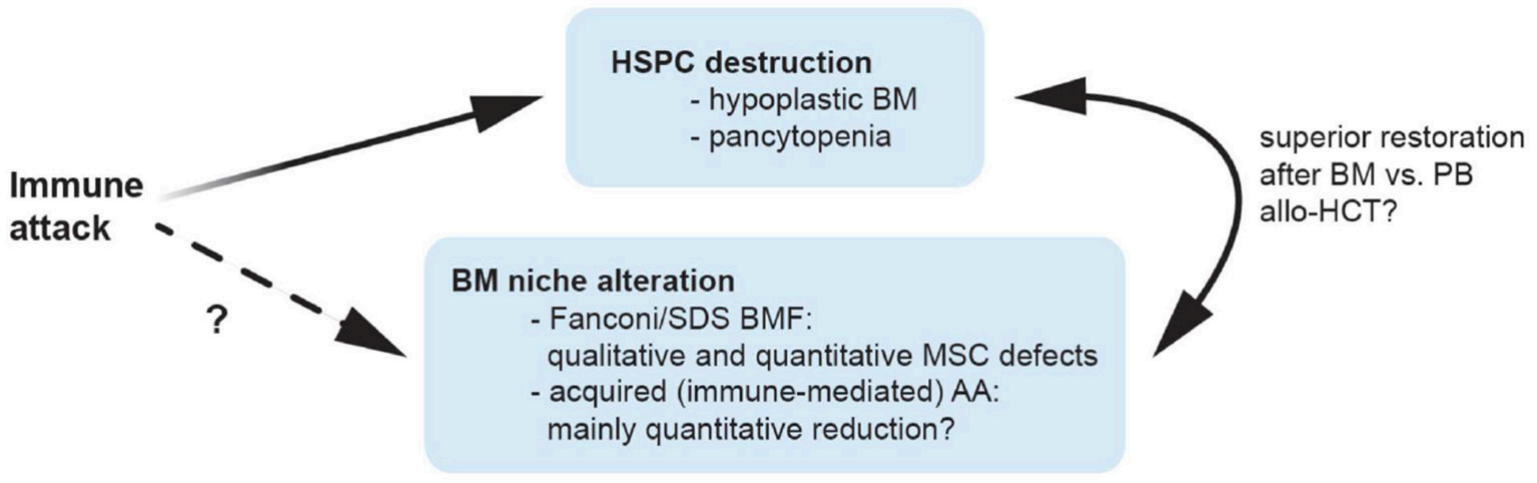
Possible mechanisms contributing to bone marrow niche modulation in aplastic anemia. Patients with aplastic anemia display not only low numbers of hematopoietic stem/progenitor cells (HSPC) but also an altered hematopoietic niche. This might result from immunologic attack and/or genetic defects impairing proliferation and survival in niche cells, or alternatively, from perturbed interactions of these with an unphysiologically diminished HSPC pool. Because of a quantitative MSC impairment in patients with acquired AA, it is tempting to speculate that bone marrow transplantations may yield better results compared to peripheral blood as stem cell source because they provide higher numbers of co-transplanted MSCs and supporting non-hematopoietic cell populations, which may promote niche reconstitution and thereby indirectly support nascent hematopoiesis in AA patients treated with allogeneic transplantations. allo-HCT, allogeneic hematopoietic cell transplantation; AA, aplastic anemia; BM, bone marrow; BMF, bone marrow failure; HSPC, hematopoietic stem and progenitor cells; MSC, mesenchymal stem cells; PB, peripheral blood; SDS, Shwachman-Diamond syndrome.

Given the deficit in HSCs/HPCs observed in patients with acquired AA or BMF, replacing these by allogeneic hematopoietic cell transplantation (allo-HCT) is an obvious treatment option (8–10) (Figure 1). Interestingly, graft rejection followed by autologous reconstitution was occasionally observed in patients with acquired AA (11) and latter attributed to immuno-suppressive effects of the conditioning therapy (12, 13). This led to the concept of treating AA patients with intensive immuno-suppressive therapies (IST) such as anti-lymphocyte globulin (14, 15) and cyclosporine (16), which nowadays represent the treatment backbone for acquired AA.

Next to the disease cause, disease severity and patient's age influence treatment choices. In symptomatic acquired AA, supportive treatment with erythrocyte and platelet transfusions and infection prevention is provided (4, 7). For severe cases, the first-line treatment is allo-HCT from a matched sibling donor in young patients (7–10) and IST in older patients without a well-matched donor (15, 17, 18). The success of allo-HCT is limited due to early and late complications, such as graft rejection, relapse due to resurgent autoimmune attack and development of graft-vs. -host-disease (GvHD), whereas disease persistence, relapse, and clonal evolution limit the success of IST. Inherited BMF are usually not responsive to IST, and besides supportive therapy, allo-HCT is the mainstay of the treatment.

The high overall response rate of about 70–80% observed in patients with acquired AA treated with IST suggests that indeed in most cases the primary mechanism inducing BM hypoplasia is of auto-immune nature (e.g., cytotoxic T cells triggering apoptosis in BM cells) (17–19). Alternative mechanisms include exposure to radiation or toxic agents such as pesticides or benzol, treatment with antineoplastic drugs, antibiotics, non-steroidal anti-inflammatory drugs, as well as active infections (e.g., with viruses such as Epstein Barr, hepatitis virus, human immunodeficiency virus, and parvovirus) (1, 2, 4). Rarely, AA is associated with lymphoproliferative neoplasms (20–22). In these cases, common denominators like particular (immuno-) genetic background or exposure to viruses and toxic environmental factors may in fact increase the risk for both diseases. On the other hand, treatment of lymphoproliferative processes may trigger auto-immunity. Possibly in a HLA-DR restricted manner, AA can co-occur as a “collateral damage” of an auto-immune process directed against the malignant lymphoid clone (21).

## Immune Dysregulation in Acquired AA

**Figure 2:**
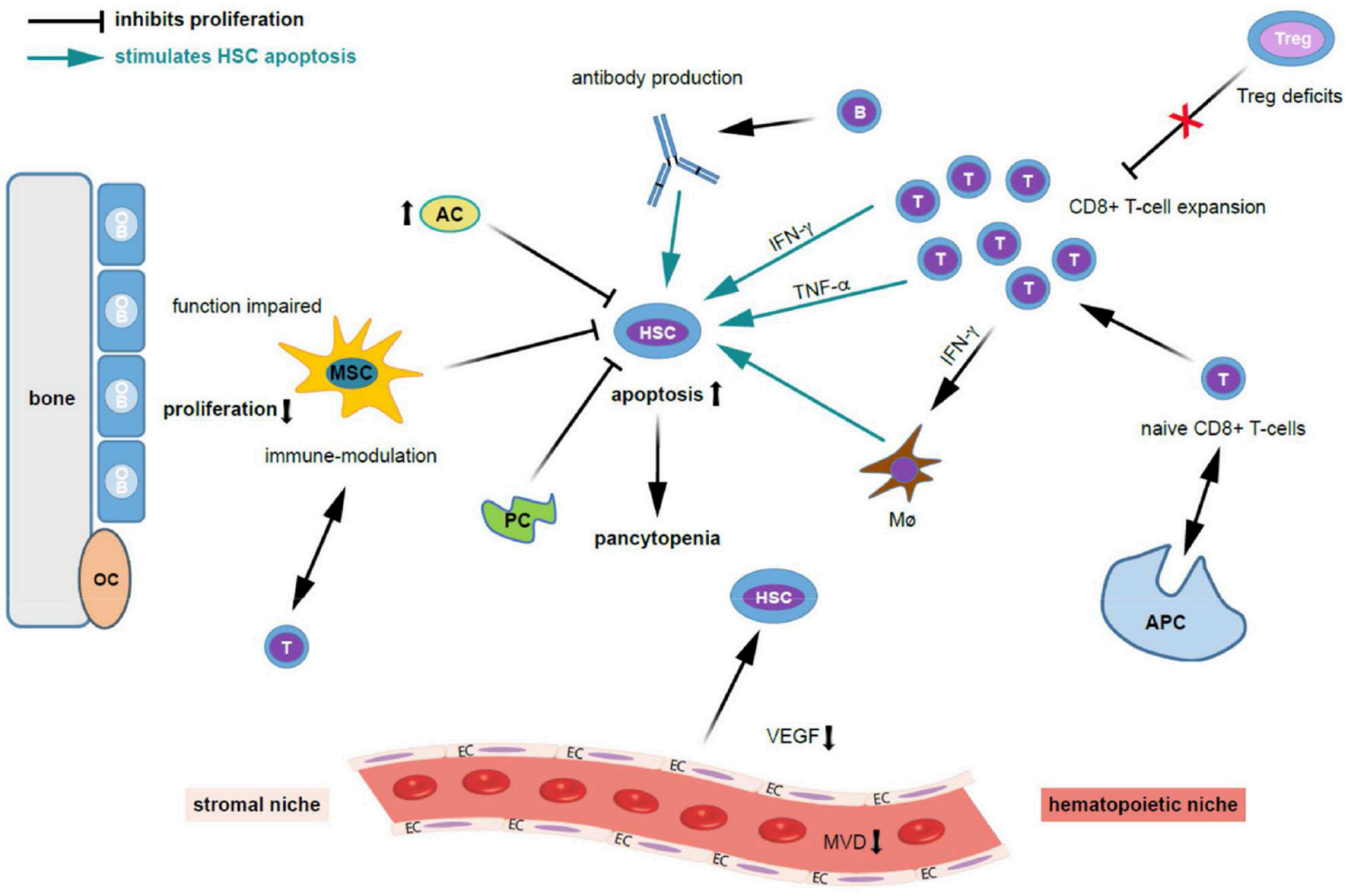
Possible mechanisms contributing to bone marrow niche modulation and immune destruction of hematopoiesis in acquired aplastic anemia. Patients with acquired aplastic anemia (AA) display not only low numbers of hematopoietic stem cells (HSC) but also an altered hematopoietic niche. On the left side of the figure the effect of stromal cells (“stromal niche”) and its interaction with HSC and on the right side the effects of the immune cells on HSC (“hematopoietic niche”) are shown. Regarding the auto-immune pathophysiology in acquired AA, antigens are presented to naive CD8^+^ T cells by antigen presenting cells (APCs), which trigger T cells to activate and proliferate. Cytotoxic T cells (a polyclonal expansion of dysregulated CD4^+^ T-cells) triggering apoptosis in bone marrow (BM) cells. Further, activated T lymphocytes induce apoptosis in HSCs and oligoclonal expansion of dysregulated CD8^+^ T-cell populations. Besides that, there is abnormal production of cytokines including interferon-gamma (IFN-γ), tumor necrosis factor-alpha (TNF- α), and transforming growth factor (TGF) which induces HSC apoptosis through Fas and the Fas ligand. These events ultimately lead to reduced cell cycling and HSC cell death by apoptosis. Quantitative and qualitative deficits of regulatory T cells (Tregs), which normally suppress auto-reactivity of other T cell populations, further stimulates T cell expansion. TNF-α-producing macrophages (Mø) in the BM were more frequent in AA patients. Further, IFN-γ-mediated HSC loss was shown to require the presence of Mø. INF-γ increases BM Mø which drives loss of megakaryocytes and HSC. The potential for IFN-γ to both directly exhaust and deplete HSCs, as well as to indirectly reduce HSC function through microenvironmental niche cells, particularly Mø, and mesenchymal stem cells (MSCs), adds complexity to the study of AA pathogenesis. Possibly, B cells, which are increased in AA patients, produce auto-antibodies against HSC. Regarding the stromal niche, impairments in osteoblastic, vascular, and perivascular HSC niches might contribute to defective hematopoiesis in patients with AA. MSC function is impaired in AA, HSCs cannot adequately proliferate, and activated T-cells are not suppressed. MSC aberrant alteration impair the maintaining of the immune homeostasis. Adipocytes (AC) are increased and pericytes are decreased (PC) and suppress hematopoiesis. Further, the microvessel density (MVD) and vascular endothelial growth factor (VEGF) expression is decreased in AA. Given the close interaction and regulatory feedback loops between resident hematopoietic and niche cells, it is not surprising that besides immune destruction, AA also associates with defects in non-hematopoietic BM microenvironment components. AC, adipocytes; APC, antigen-presenting cell; HSC, hematopoietic stem cell; EC, endothelial cells; INF-γ, interferon-gamma; MVD, microvessel density; Mø, macrophages; MSC, mesenchymal stem cells; OB, osteoblasts; OC, osteoclasts; PC, pericytes; TNF-α, tumor necrosis factor-alpha; VEGF, vascular endothelial growth factor.

The cause of acquired AA was not clear for many years. While initially toxic effects were postulated as the reason of a quantitative HSC defect, nowadays autoimmune processes are considered mainly responsible for acquired AA occurring in the absence of a positive medical history of predisposing drugs, toxic agents or infections (1–4) (Figure 1). In fact, while several pathomechanisms have been proposed, the greatest proportion of cases is likely due to uniform T-cell mediated auto-immunity and marrow destruction leading to defective, nearly absent hematopoiesis. Consistently, activated T lymphocytes were observed to induce apoptosis in HSCs (1–4) and oligoclonal expansion of dysregulated CD8^+^ T-cell populations demonstrated in *ex vivo* BM models of AA patients (Figure 2) (19, 23, 24). Furthermore, increases in T-helper 17 (Th17) cells, the effector cells which produce the pro-inflammatory cytokine interleukin-17 (IL-17), were found in peripheral (PB) and BM of AA patients (1, 3, 25). Disease activity associated positively with enhanced numbers of Th17 and interferon (IFN)-γ-producing cells, and negatively with regulatory T cells (Treg) populations known to suppress auto-reactivity of other T-cell populations to normal tissue including the BM environment and HSCs. Indeed, especially Tregs from the BM of patients with AA were found to show pronounced quantitative as well as qualitative defects (25).

Further, BM lymphocytes from AA patients were shown to effectively inhibit hematopoietic cells from healthy donors in co-culture experiments (23). While the antigenic exposure leading to a polyclonal expansion of dysregulated CD4^+^ T-cells and, respectively, the antigens targeted by T-cells on HSCs remain unknown, the subsequent overproduction of pro-inflammatory cytokines such as IFN-γ as well as tumor necrosis factor (TNF)-α are likely involved in disease pathogenesis (26–29) (Figure 2). In experimental models, *in vitro* addition of anti-IFN-γ to BM cells from AA patients enhanced the amount of hematopoietic colonies, while the same treatment did not affect cultures from healthy BM cells (26–30). Mechanistically, IFN-γ binds to IFN-γ receptors to modulate the signal transducer and activator of transcription (STAT) and suppressor of cytokine signaling 2 (SOCS2) pathways and influence proliferation and stemness (31). In a very recent study by Sun et al. in a mouse model infusion of TNF-α^−/−^ donor lymph node (LN) cells into CByB6F1 recipients or injection of FVB LN cells into TNF-αR^−/−^ recipients both induced BM failure, with concurrent marked increases in plasma IFN-γ and TNF-α levels (32). In TNF-α^−/−^ recipients, BM damage was attenuated, suggesting that TNF-α of host origin was essential for immune destruction of hematopoiesis. Depletion of host macrophages before LN injection reduced T-cell IFN-γ levels and reduced BM damage, while injection of recombinant TNF-α into FVB-LN cell-infused TNF-α^−/−^ recipients increased T-cell IFN-γ expression and accelerated BM damage. Compared to healthy donors, TNF-α-producing macrophages in the BM were more frequent in AA patients. They concluded that TNF-α from host macrophages and TNF-αR expressed on donor T cells are critical in the pathogenesis of murine immune-mediated BM failure. Further, compared to healthy donors the frequency of TNF-α-producing CD16^+^CD68^+^ macrophages in the BM is higher in AA patients (32).

Interestingly, in a recently published mouse model of severe AA, IFN-γ-mediated hematopoietic stem cell loss was shown to require the presence of macrophages (33). Despite loss of other myeloid cells and HSCs, IFN-γ was also required for BM macrophage persistence. T cell activation or IFN-γ production in the BM was not impaired by depleting macrophages or terminating IFN-γ signaling specifically in macrophages, but instead rescued HSC numbers and reduced mortality (33). Thus, macrophages rather act as sensors of IFN-γ and are not required for induction of IFN-γ in AA. Macrophage depletion rescues thrombocytopenia, increases BM megakaryopoiesis, preserves platelet-primed stem cells, and increases the platelet-repopulating capacity of transplanted HSCs.

## The Bone Marrow Microenvironment in AA

In murine models, acquired AA associates with a shortage of HSCs (34). In patients with AA, PB and BM show drastically reduced levels of CD34^+^ cells and, respectively, of so-called long-term culture initiating cells (LT-CIC), which are the functional equivalent of populations enriched for HSCs in humans (35–38). A critical factor sustaining a healthy HSCs/HPCs production is the BM microenvironment (39, 40) (Table 1). Important components thereof are BM stromal cells, the extracellular matrix and local cytokine gradients (40, 49). The hematopoietic and non-hematopoietic elements of the BM closely interact with each other thereby sustaining and balancing hematopoiesis and hematopoietic output (Figure 2). There is clear evidence that the BM microenvironment contains specialized HSC niches that include endothelial, perivascular and endosteal cells and provide key signals that regulate survival, quiescence, self-renewal and differentiation in HSCs (7).

**Table 1:** Selection of relevant studies about bone marrow microenvironment in aplastic anemia.

**Type of study/ species**	**Main findings**	**Mechanisms**	**References**
Mice	Role of macrophages in AA	- Depleting macrophages or abrogating IFN-γ signaling in macrophages did not impair T-cell activation or IFN-γ production in the BM but rescued HSCs - Macrophages are not required for induction of IFN-γ in SAA and rather act as sensors of IFN-γ - Macrophage depletion rescued thrombocytopenia, increased BM megakaryocytes, preserved platelet-primed stem cells, and increased the platelet-repopulating capacity of transplanted HSCs	(33)
Mice, human	TNF-α from host macrophages and TNF-αR expressed on donor T cells are critical in the pathogenesis of murine immune-mediated BM failure - AA patients have higher frequencies of TNF-α-producing CD16^+^CD68^+^ macrophages in the BM than do healthy donors	- Infusion of TNF-α^−/−^ donor LN cells into CByB6F1 recipients mice or injection of FVB LN cells into TNF-αR^−/−^ recipients both induced BM failure, with marked increases in plasma IFN-γ and TNF-α levels - In TNF-α^−/−^ recipients, BM damage was attenuated, suggesting that TNF-α of host origin was essential for immune destruction of hematopoiesis - Depletion of host macrophages before LN injection reduced T-cell IFN-γ levels and reduced BM damage, while injection of recombinant TNF-α into FVB-LN cell-infused TNF-α^−/−^ recipients increased T-cell IFN-γ expression and accelerated BM damage - Infusion of TNF-αR^−/−^ donor LN cells into CByB6F1 recipients reduced BM T-cell infiltration, suppressed T-cell IFN-γ production, and alleviated BM destruction - In AA patients, TNF-α-producing macrophages in the BM were more frequent than in healthy donors	(32)
Mice	ROS generation is associated with BM failure in AA	- Increased ROS and disruption of hematopoietic niche under aplastic stress - Decline of stromal components and deregulation of Notch-1/ Jagged-1 signaling axis in aplastic marrow - Altered DNA methylation and H-3 phosphorylation status associated with redox imbalance in aplastic marrow	(41)
Human	VEGF-Notch signaling pathway	- Lower expression of VEGF, VEGFR, Notch-1, Jagged1, Delta-like1, and hes1 was revealed in AA BM tissues and AA MSCs - The intervention of DAPT (a γ-secretase inhibitor) significantly inhibited proliferation, and promoted the apoptosis and adipogenic differentiation of AA MSCs, while VEGF intervention exhibited opposite results - The proliferation, migration, and angiogenesis of HUVECs were significantly promoted by normal BM-MSCs, while inhibited by VEGF/Notch-1 shRNA transfected BM-MSCs	(42)
Human, mice	Effect of CD106 and NF-κB in BM failure of AA	- BM-MSCs from AA patients exhibited downregulation of the CD106 gene (VCAM1) and low expression of CD106 *in vitro* - CD106^+^ MSCs from both AA patients and healthy controls had increased potential for *in vitro* capillary tube-like formation and *in vivo* vasculogenesis compared with CD106^−^ MSCs - CD106^+^ MSCs from both AA patients and healthy controls more strongly supported *in vitro* growth and *in vivo* engraftment of CD34^+^ cells in NOD/SCID mice than CD106^−^ MSCs - Expression of NF-κB was decreased in AA MSCs, and NF-κB regulated the CD106 gene (VCAM1) which supported hematopoiesis	(43)
Human	Vascular and perivascular niches are numerically restored, but the endosteal niche remains numerically impaired in patients with AA after allo-HCT	- Levels of VEGF, but not donor-derived BM-MSCs, may correlate with the restoration of BM niches	(44)
Human	AA is associated with impaired hematopoietic stem cell niches	- Patients with AA showed markedly fewer endosteal cells, vascular cells, and perivascular cells compared with controls	(45)
Human	The biological characteristics of AA MSC are different from those of control MSC and their *in vitro* haemopoiesis -supporting ability is significantly reduced	- AA MSC presented typical morphology and distinctive mesenchymal markers, stromal formation was reduced, with 50% of BM samples failing to produce adherent layers - Their proliferative and clonogenic capacity was markedly decreased and the ability to sustain haemopoiesis was significantly reduced, as assessed by total cell proliferation and clonogenic potential of HSC	(46)
Human, mice	BM-MSCs from patients with AA do not have impaired functional and immunological properties, suggesting that they do not contribute to the pathogenesis of the disease	- MSCs cultures can be established from the BM of AA patients and display the same phenotype and differentiation potential as their counterparts from normal BM - MSCs from AA patients support the *in vitro* homeostasis and the *in vivo* repopulating function of CD34^+^ cells, and maintain their immunosuppressive and anti-inflammatory properties	(47)
Human	Gene expression profile of BM-MSCs confirmed the abnormal biological properties and provided significant evidence for the possible mechanism of the destruction of the BM microenvironment in AA	- BM-MSCs from AA patients showed aberrant morphology, decreased proliferation and clonogenic potential and increased apoptosis compared to controls - BM-MSCs from AA patients were susceptible to be induced to differentiate into adipocytes but more difficult to differentiate into osteoblasts - A large number of genes implicated in cell cycle, cell division, proliferation, chemotaxis and hematopoietic cell lineage showed markedly decreased expression in BM-MSCs from AA patients - Conversely, more related genes with apoptosis, adipogenesis and immune response showed increased expression in BM-MSCs from AA patients	(48)

*AA, aplastic anemia; allo-HCT, allogeneic hematopoietic cell transplantation; BM, bone marrow; BM-MSCs, bone marrow mesenchymal stem cells; HSCs, hematopoietic stem cells; HUVECs, human umbilical vein endothelial cells; IFN-γ, interferon-gamma; LN, lymph node; MSC, mesenchymal stem cells; ROS, reactive oxygen species; SAA, severe aplastic anemia; shRNA, short hairpin RNA; TNF-α, tumor necrosis factor-alpha; VEGF, vascular endothelial growth factor; VEGFR, vascular endothelial growth factor receptor*.

Given the close interaction and regulatory feedback loops between resident hematopoietic and niche cells, it is not surprising that AA also associates with defects in non-hematopoietic BM microenvironment components. As such, patients with AA showed markedly fewer endosteal cells, vascular cells, and perivascular cells compared with controls and loss of non-hematopoietic podoplanin-positive stromal cells was reported in a SAA mouse model (33). In a further study, Chatterjee et al. examined the mechanisms underlying chemotherapeutics mediated BM aplasia (41). They demonstrated that ROS (reactive oxygen species) generated in response to chemotherapy treatment indeed negatively impact the hematopoietic niche (by deregulation of microenvironment related Notch-1 signaling) and thereby alter the epigenetic status of the HSCs leading to devastating HSC damage. They thus proposed that anti-oxidant based therapeutic strategies may be used to circumvent such adverse effects on hematopoiesis.

Impairments in osteoblastic, vascular, and perivascular HSC niches might contribute to defective hematopoiesis in patients with AA (8, 44). Several studies have reported abnormal function and disordered components of the BM microenvironment in patients with AA and BMF (50–53). For example, long-term cultures of BM stromal cells from patients with AA were shown to less robustly support hematopoiesis (44, 51). Mesenchymal stem cells (MSCs) are multipotent stromal cells that can differentiate into a variety of cell types: Namely adipocytes (fat cells which give rise to marrow adipose tissue), chondrocytes (cartilage cells), osteoblasts (bone cells), and myocytes (muscle cells) (3). Compared to healthy donors, MSCs derived from patients with AA showed aberrant morphology, decreased proliferation and clonogenic potential, increased apoptosis and a propensity to differentiate into adipogenic at the expense of osteogenic lineages (46, 48, 54–57), even if significant heterogeneity among individual patient samples was observed. Consistently, transcriptome analyses performed on these cells revealed altered expression especially of genes involved in cell proliferation, cell division, cell cycling, chemotaxis, hematopoietic cell interactions adipogenesis, and immune response in AA vs. healthy controls (43, 48).

In contrast, more recent studies reported MSCs derived from patients with acquired AA to not differ from those collected from healthy controls (47, 58). In particular, MSCs from acquired AA patients were shown to form adequate HSC niches *in vivo*, comparable to those resulting from healthy control MSCs (58). These controversial results might be due to (a) heterogeneity among individual patients (since in all studies a limited number of patients was analyzed), (b) technical differences among the employed assays and/or (c) potentially inclusion of patients with unrecognized congenital genetic defects in the analysis, since—especially in the older studies—advanced genetic testing was perhaps not uniformously used to distinguish between patients with immune-mediated acquired AA and aplasia resulting from congenital genetic syndromes. In latter, stromal and other non-hematopoietic microenvironmental cells carry also themselves the congenital genetic lesion and might be thus affected by it. Indeed a comprehensive comparison of MSCs derived from 18 patients with Fanconi anemia (*n* = 18) and age-matched healthy controls (*n* = 15) revealed increased spontaneous chromosomal fragility leading to precocious senescence and significantly reduced survival when compared to MSCs derived from age-matched healthy donors (59). These data were further confirmed in a subsequent study from Cagnan et al. which documented decreased proliferation, increased ROS levels and an arrest in G2 following DEB (Diepoxybutane) treatment in MSCs from 10 Fanconi anemia patients, with especially absent transforming growth factor (TGF)- β secretion and elevated senescence levels in the FANCD2 mutated cases (60). Analysis of MSCs from Shwachman-Diamond syndrome (SDS) patients, another major subgroup of congenital BMF, also revealed profound functional defects and failed to recreate a BM niche in an *in vivo* heterotopic ossicle model (61). When compared to healthy donor derived MSCs, these SDS-MSCs especially displayed a marked decrease in vascular endothelial growth factor (VEGF) expression and defective ability to form correct vascular networks, capillary tubes and vessels (61). In fact, using a genetic model of pre-leukemic SDS Zambetti et al. recently showed that perturbation of the MSC compartment induced mitochondrial dysfunction, oxidative stress and activation of the DNA damage responses in HSCs/HPCs (62), and suggested inflammation mediated niche-dependent genotoxic stress in HSCs/HPCs as mechanism promoting leukemogenesis in SDS. Together, these data suggest that the observed MSC impairment observed in acquired immune-mediated AA may be predominantly of quantitative nature while patients with BMF associated with Fanconi or SDS also display important qualitative MSC defects (Figure 1). It remains to be determined whether the enhanced risk of progression to malignant hematopoietic diseases (which is much higher in Fanconi or SDS syndromes, when compared to acquired immune-mediated AA) is in part also mediated by such qualitative changes in microenvironment cells.

## Superiority of BM vs. Peripheral Blood Stem Cells (PBSC) Allogeneic Transplantation in Patients with Acquired AA

BM or mobilized PB derived HSCs/HPCs can both be used for allogeneic HCT therapies. Interestingly, unlike in most other conditions routinely treated by allo-HCT, unmanipulated BM is recommended as stem cell source in AA patients referred to allo-HCT. In a study by Bacigalupo et al. (63), BM vs. PB was examined as stem cell source in 1886 patients with AA. Patients receiving BM had a significant survival advantage compared to patients receiving PB as stem cell source. This advantage was statistically significant in both, patients aged 1–19 years (90% vs. 76% *P* < 0.00001) as well as in patients aged over 20 years (74% vs. 64%, *P* = 0.001) (63). The advantage for BM as stem cell source compared to PBSC was maintained above the age of 50 years (69% vs. 39%, *P* = 0.01). Patients with PB as stem cell source had a higher incidence of acute and chronic graft-vs. -host disease. This study reinforced that BM should be the preferred stem cell source for matched sibling transplants in acquired AA, irrespectively of the patients' age. A main reason for this recommendation is considered the increased risk of chronic GvHD after use of PB stem cells (which should be especially avoided in patients transplanted for AA as a non-malignant disease). Despite an earlier engraftment was observed with the use of PB stem cells, a joint EBMT/CIBMTR retrospective analysis suggests inferior outcome with the use of PBSC in this disease, particularly in younger patients (64). More recently, the survival advantage for BM grafts was confirmed across all age groups (65). A BM stem cell dose of at least 3 × 10^8^ MNC/kg or 2 × 10^6^ CD34/kg should be given, as a low stem cell dose increases the risk of graft failure (66). PB is an alternative stem cell source only in case of contraindications to a BM harvest, unwillingness of the donor to donate BM, or in case of second transplants after graft failure.

Given the above mentioned quantitative MSC impairment in patients with acquired AA, it is tempting to speculate that BM transplantations may yield better results because they provide higher numbers of co-transplanted MSCs and supporting non-hematopoietic cell populations, which may promote niche reconstitution and thereby indirectly support nascent hematopoiesis in AA patients treated with allogeneic transplantations (Figure 1). Indeed, as shown by De Felice et al. (67) in contrast to BM grafts which contained mesenchymal progenitors that could be further stimulated by granulocyte colony-stimulating factor (G-SCF) treatment, no mesenchymal progenitors activity was detected using the same functional assays among cells collected from the peripheral blood of healthy donors, regardless of G-CSF stimulation (67). The authors concluded, that BM could be a more useful HSC source for transplant as well as an MSC source because MSC progenitors were undetectable in PB before and after G-CSF stimulation. Several mechanisms may influence the restoration of the BM niche in case functional non-hematopoietic progenitor cells are transplanted next to HSCs/HPCs with BM vs. PB transplants (68–70): (1) Donor-derived BM-MSCs (71) may engraft and then differentiate to niche cells, vascular endothelial cells and perivascular cells after HCT; (2) CD34^+^ progenitor cells from the transplanted graft may generate endothelial cells in an appropriate host environment (72); (3) Numerous cytokines secreted by HSCs/HPCs and transplanted niche cells may influence the microenvironment and enhance BM stem cell homing and subsequent hematopoiesis (69, 71, 73).

Interestingly, data from AA patients receiving allo-HCT (most patients received BM together with PBSCs as stem cell source) showed that in spite of successful hematopoietic engraftment after allo-HCT, BM-MSCs remained host-derived (44). However, co-transplanted donor MSCs and non-hematopoietic populations contained in BM allo-transplants could perhaps transiently support the regeneration of the niche (and therefore of the hematopoietic compartment) in AA patients with quantitative niche defects. Alternatively, they might provide additional immune suppressive effects that improve the course of the disease (3).

AA can be cured or ,respectively, ameliorated by allo-HCT or IST (9, 10, 15, 18). How an allo-HCT is influencing the BM microenvironment and especially the HSC niche, is not understood in detail. Wu et al. (44) analyzed the HSC niche components changes by immunohistochemistry (CD34, CD146, and osteopontin) before allo-HCT and at 1, 2, 3, 6, and 12 months after allo-HCT (44). A significant increase in the number of cellular elements in the BM niche, including vascular and perivascular cells could be shown in patients with acquired AA (*n* = 52) after allo-HCT (44). Regarding endosteal cells, no relevant changes could be found. To get more insight into the restoration of the BM niche after allo-HCT, the origin of BM-MSCs and the expression of cytokines in BM plasma were examined. At 1–12 months after transplantation, BM-MSCs were indeed derived from the host, not the donor. Further, after allo-HCT levels of VEGF significantly rose (44). Taken together, in patients with AA after allo-HCT vascular and perivascular niches are numerically restored, but the endosteal niche remains numerically (quantitatively) impaired. Allo-HCT from BM cells thus improves reconstitution of some (perhaps VEGF-dependent) but not all niches. Of note, a systematic comparison between BM and PB allo-transplants was not yet performed, thus the role of co-transplanted non-hematopoietic BM cells for these effects remains elusive. The restoration of BM niches may be the result of high VEGF levels, but not of donor-derived BM-MSCs.

Postnatal vasculogenesis during physiological and pathological neovascularization occurs by endothelial progenitor cells with BM origin (70). CD34^+^ endothelial/hematopoietic BM derived progenitors were shown to promote vasculogenesis and osteogenesis during bone healing (74), suggesting that also in the allo-HCT transplant setting they might promote niche reconstruction. However, as shown by Wu et al. (45) the osteoblastic niche-forming cells remained at low numbers after allo-HCT. The most likely explanation for the specific differences among niche cells is that transplanted CD34^+^ cells are localized to distinct locations and influence niche restoration. Cell-intrinsic and niche intrinsic variables influences the relationship of transplanted primitive hematopoietic cells to anatomical components of the trabecular niche (75).

When BM microvessel density (MVD) was compared using immunohistochemical staining for CD34, patients with severe AA showed significantly lower MVD compared with patients with non-severe AA, and ,respectively, healthy controls (50) (Figure 2). The formation of the vasculature is mediated through VEGF secretion by CD34^+^ cells (76–78). A further study confirmed the lower BM MVD in patients with newly diagnosed AA and additionally demonstrated reduced VEGF serum levels in these cases compared to healthy controls (50). Moreover, response to therapy (IST or allo-HCT) was shown to associate with increase of both VEGF and MVD (50). In summary, AA is associated with reduced angiogenesis and reduced VEGF expression, which might contribute to disease pathogenesis. The secretion of VEGF from successfully engrafted hematopoietic - and perhaps also non-hematopoietic—BM cells after allo-HCT is likely to support the regeneration of the impaired BM environment in AA patients. In a study by Deng et al. the role of the VEGF-Notch signaling pathway on MSC was examined in AA (42). They found a significantly lower expression of VEGF, VEGFR, Notch-1, Jagged1, Delta-like1, and hes1 in AA BM tissues and AA MSCs compared to healthy controls. The activation of VEGF-Notch signaling promoted the proliferation and adipogenic differentiation, and inhibited the apoptosis of AA MSCs. They concluded that the activation of VEGF-Notch signaling pathway may be a potential therapeutic target for AA. In the embryo, HSCs are formed from endothelial cells upon activation of the VEGF-Notch pathway (79–81). Regulatory loops by which VEGF controls the survival of HSCs have been described (78), as well as cross-talk between hematopoietic, endothelial and mesenchymal cells in embryonic as well as adult hematopoietic environments. It remains unclear whether the observed decreased BM vascularization in AA is instrumental in the bone marrow failure process or rather another consequence of the profoundly disturbed hematopoiesis with reduced content of cytokine producing cells. Nevertheless, such a strongly impaired BM microenvironment is likely to sustain hematopoietic defects and furthermore delay hematopoietic regeneration even after the causative agent was removed (e.g., by IST or replacement of the altered immune system by allogeneic transplantation).

Taken together, a disturbed microenvironment with abnormal functioning MSCs is, next to BM hypoplasia, a further hallmark in patients with acquired AA. MSCs have been utilized in the settings of therapy for other disorders due to their immunomodulatory and proliferative functions; e.g., steroid-refractory gastrointestinal graft-vs. -host disease. Translation of MSC therapy to AA has been relatively limited. Preliminary studies have attempted to use MSCs as an adjunct to allo-HCT to improve engraftment or as primary, monotherapy of AA (82, 83). However, larger studies are needed to evaluate the utility of MSCs further. In a recent study by Yue et al. in patients with SAA, haploidentical-HCT from related donors was performed together with culture-expanded allogeneic bone marrow-derived MSCs, which were infused on day 0 and day +14 (84). All patients achieved sustained, full donor chimerism, and the median time of myeloid recovery and platelet engraftment was 13 days, respectively. MSCs and other non-hematopoietic cells present in the BM vs. PB as stem cell source, may contribute to niche regeneration post-transplantation by (a) providing transient VEGF and cytokine support, (b) enhancing immune suppression and possibly (but less probable) by (c) providing stable niche engraftment of donor-derived niche cells. Considering that multiple progenitor cells, as well as HSCs are enriched in transplanted PBSC and BM (68–70, 85) there may be several possible mechanisms to explain the restoration of the BM niche which include transient engraftment of cytokine producing non-hematopoietic BM cells that promote niche restoration and thereby facilitate hematopoietic regeneration (69–72).

## Conclusion

The cross-talk between the microenvironment and the defective hematopoietic compartment may significantly contribute to the disrupted hematopoiesis and delayed hematopoietic regeneration commonly observed in AA patients. There are several *in vitro* and *in vivo* studies indicating quantitative as well as qualitative mesenchymal stromal alterations in patients with AA and, respectively, genetic BMF, which may be pathogenetically involved in the clinical evolution of the disease. Further, studies are needed to examine the importance of microenvironment changes in the acquired AA pathogenesis, progression and response to therapy. A better understanding of these processes may unravel novel niche-based therapeutic intervention possibilities that ameliorate the clinical course of AA and/or improve response to therapy by augmenting niche support.

## Author Contributions

All authors listed have made a substantial, direct and intellectual contribution to the work, and approved it for publication.

### Conflict of Interest Statement

The authors declare that the research was conducted in the absence of any commercial or financial relationships that could be construed as a potential conflict of interest.
